# MR-linac: role of artificial intelligence and automation

**DOI:** 10.1007/s00066-024-02358-9

**Published:** 2025-01-22

**Authors:** Serena Psoroulas, Alina Paunoiu, Stefanie Corradini, Juliane Hörner-Rieber, Stephanie Tanadini-Lang

**Affiliations:** 1 Department of Radiation Oncology, University Hospital Zurich, University of Zurich, Rämistrasse 100, 8091 Zurich, Switzerland; 2 Department of Radiation Oncology, University Hospital, LMU Munich, Munich, Germany; 3 Department of Radiation Oncology, Heidelberg University Hospital, Heidelberg, Germany; 4 Heidelberg Institute of Radiation Oncology (HIRO), Heidelberg, Germany; 5 National Center for Tumor Diseases (NCT), Heidelberg, Germany; 6 Clinical Cooperation Unit Radiation Oncology, German Cancer Research Center (DKFZ), Heidelberg, Germany; 7 Department of Radiation Oncology, University Hospital Düsseldorf, Düsseldorf, Germany

**Keywords:** Artificial intelligence, MR-guided radiation therapy, Automation, Imaging biomarkers, Intrafractional motion management

## Abstract

The integration of artificial intelligence (AI) into radiotherapy has advanced significantly during the past 5 years, especially in terms of automating key processes like organ at risk delineation and treatment planning. These innovations have enhanced consistency, accuracy, and efficiency in clinical practice. Magnetic resonance (MR)-guided linear accelerators (MR-linacs) have greatly improved treatment accuracy and real-time plan adaptation, particularly for tumors near radiosensitive organs. Despite these improvements, MR-guided radiotherapy (MRgRT) remains labor intensive and time consuming, highlighting the need for AI to streamline workflows and support rapid decision-making. Synthetic CTs from MR images and automated contouring and treatment planning will reduce manual processes, thus optimizing treatment times and expanding access to MR-linac technology. AI-driven quality assurance will ensure patient safety by predicting machine errors and validating treatment delivery. Advances in intrafractional motion management will increase the accuracy of treatment, and the integration of imaging biomarkers for outcome prediction and early toxicity assessment will enable more precise and effective treatment strategies.

## Introduction

Artificial intelligence (AI) has led to remarkable progress in radiation therapy in the past 5 years, particularly through the successful implementation of automated processes [1, 2]. One notable achievement is the automatic delineation of organs at risk (OARs), which is nowadays already used by many departments in daily clinical practice [3, 4]. It presents a significant time-saving benefit while ensuring more consistent and precise delineations. Another achievement of an AI application in clinical practice is the automatic generation of treatment plans, a development that not only increases consistency but also substantially reduces the time required, particularly for standard cases [5, 6]. Some pioneering groups have even demonstrated fully automated processes encompassing OAR delineation, target volume delineation, planning, and quality assurance [7–9]. However, such fully automated processes are not yet commercially available and are not routinely used. Although the exploration of AI in decision support for radiotherapy is ongoing, with promising research outcomes, it is noteworthy that these advancements are currently in the research stage and await clinical implementation.

Magnetic resonance (MR)-guided linear accelerators (MR-linacs) have the potential to significantly improve treatment accuracy by providing real-time imaging with high soft tissue contrast during the treatment sessions. This enables the adaptation of treatment plans to the anatomy of the day and real-time motion management [10]. Several groups have shown that MR-guided radiotherapy (MRgRT) better spares normal tissues and reduces side effects [11–16].

Excellent clinical results have especially been reported for moving or critically located tumors adjacent to radiosensitive OARs, such as prostate, rectal, pancreatic, hepatic, adrenal, and ultracentrally located lung tumors [16–22]. MRgRT holds the potential to allow for safe dose escalation in close proximity to vulnerable normal tissues [17, 23–26]. In line with the abovementioned results, the first recently published randomized phase III trial comparing MR- to computed tomography (CT)-guided stereotactic radiotherapy reported both reduced acute physician-scored toxic effects as well as superior patient-reported quality of life when applying MRgRT for prostate cancer [27].

Although MRgRT presents notable advantages in terms of improved visualization and treatment accuracy, it is crucial to recognize the accompanying challenges. A primary obstacle lies in the laborious and time-consuming nature of MR-guided radiotherapy procedures. Key tasks like delineation and treatment planning are predominantly manual, involving clinicians or physicists. Consequently, this manual intervention frequently extends treatment durations, typically spanning between 30 and 70 min [18, 28, 29].

While the advantages of MR-guided radiotherapy are evident, the complexity and real-time nature of the imaging data present new challenges. The need for rapid decision-making and adaptive planning requires new solutions, and this is where AI and automation will play a crucial role in the future. AI algorithms can analyze large datasets derived from MR imaging, providing real-time insights into anatomical variations and facilitating adaptive treatment strategies. Automation has the potential to further streamline this process by integrating AI into the treatment workflow, ensuring seamless communication between imaging, planning, and delivery systems.

**Fig. 1:**
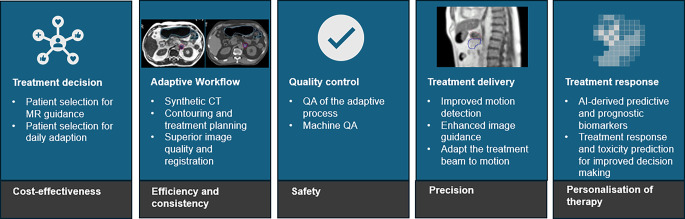
Potential applications of artificial intelligence (*AI*) in the MR-linac workflow: the potential of AI in magnetic resonance-guided radiotherapy for selecting patients, supporting the adaptive process, ensuring the quality of the treatment, managing motion during treatment delivery, and deriving predictive and prognostic biomarkers

This article summarizes the benefits of integrating AI and automation in MR-linacs, emphasizing the potential to elevate treatment precision, safety, cost effectiveness, efficiency, and personalization (Fig. 1). Automating the interplay between MR imaging, treatment planning, and delivery will make the MR-linac technology more powerful to benefit more patients.

## AI as decision support in MRgRT

The availability of an adaptive workflow is one of the main advantages of an MR-linac over other radiotherapy techniques but comes at a high price concerning resources and personnel. During an adaptive treatment, radiation oncologists, medical physicists, and radiation therapy technicians all need to be present and work in parallel, when possible, or in sequence if required. While AI will help to streamline these processes in the future and significantly increase the number of slots available per working day, patient selection for adaptive workflows remains an important factor. Strategies for how to select patients for MRgRT have been reported by different institutes. As clinical experience with these systems increases, screening becomes more complex, and many factors need to be considered to ensure the right workflow is selected [30]. As a minimum requirement, the tumor size and the distance between the tumor and organs at risk are usually considered [31], together with identifying anatomical sites where CT density is rather homogeneous and better soft tissue contrast for daily adaptation can therefore be obtained with MRI than with conebeam CT (CBCT). However, this information is still mainly based on clinical experience and may suffer from biases. Health economic analyses have been suggested to provide an objective basis for decision-making in patient selection [32] but these are not yet standard in clinical practice.

In the context of patient selection, AI could empower decision-making if we were able to fully understand what the data reveal to us. As MR-linacs routinely take patient images during the course of the treatment, ideally longitudinal studies between pretreatment MRI, online MRIs, and posttreatment MRIs would allow to identify biomarkers predictive of an outcome [33] (discussed below in “AI to enhance intrafractional motion management”). The feasibility of such longitudinal studies during the course of treatment has already been demonstrated [34–36]. In the context of patient selection, some biomarkers could eventually be searched for at the time of diagnosis to identify the patients who would more likely benefit from MR-linac treatments. Subsequently, during treatment, adaptation would also be able to account for the predicted response of the tumor and/or healthy tissue. Furthermore, AI could facilitate the integration and assessment of clinical, pathological, genomic, and imaging big data available prior to treatment, to select for patients who would profit most from MRgRT [1, 37]. This development, however, is still far from clinical practice.

One big challenge will of course be the characteristics of the training dataset itself, as patients are already selected for treatment now based on specific parameters. Therefore, the resulting dataset, with MRI images taken at all stages of the treatment, will have a selection bias that needs to be accounted for. Data sharing between different institutes would be advisable but complicated in practice. Additionally, the image quality (which is not the same between a diagnostic MRI and a hybrid MRI) can make it difficult to identify biomarkers on images taken on an MR-linac; we refer the reader to the chapter “AI-derived imaging biomarkers for outcome prediction and early toxicity assessment” of this review for an overview of such challenges.

## AI for automatizing the adaptive workflow

Significant progress has been made in generating synthetic CTs from MR images, which is crucial for transitioning to MRI-only radiotherapy workflows. Currently, online MRgRT still requires an initial CT scan during treatment planning to obtain electron density information for accurate dose calculations. On subsequent treatment days, standard practice involves registering the original CT with new MR scans using deformable image registration (DIR) or a combination of DIR and bulk density override. Replacing the CT scan with a precise synthetic CT can eliminate additional radiation exposure and reduce registration uncertainties [38, 39].

Recent studies have demonstrated the feasibility of generating synthetic CTs using deep learning for both low- and high-field MR-linacs [40–43]. These approaches have shown high dosimetric accuracy for treating pelvic [43], abdominal, liver [44], and breast cancers [42]. For online adaptive treatments, rapid synthetic CT generation is essential to mitigate interfractional patient and internal anatomy deviations. Deep learning methods have achieved this, generating synthetic CTs in as little as 25 s using CPUs and within 6 s using GPUs [43, 45]. This technology is nearing clinical readiness, combining both excellent accuracy and efficiency.

Accurate delineation of OAR and target volumes is crucial for treatment planning and adaptation but is often time consuming and subject to interobserver variability [46]. While several commercial solutions exist for CT-based automatic contouring of OARs and elective lymph node levels, MRI-based solutions are needed for oMRgRT. Studies have shown high accuracy in automatic deep learning-based contouring for 1.5T MR images in the pelvis, head, neck, and abdomen [47–49]. However, limited data are available for low-field MR images (0.35T), with only a few studies investigating automatic contouring for the pelvis and abdomen [50–53]. With MRI’s superior soft tissue contrast and the increasing installation of MR-linacs, a clinically approved solution for OAR delineation is anticipated soon. Delineating the gross target volume remains more challenging due to greater interobserver variability, necessitating further research and data to improve accuracy.

Artificial intelligence-based automatic treatment planning has shown promise for improving data consistency and standardizing treatment processes. While automatic treatment planning in conventional radiotherapy has been extensively researched [54–56], its application in online MRgRT remains less explored. The need for rapid results in online adaptive procedures makes automatic treatment planning suitable for real-time plan generation. Künzle et al. developed an autonomous treatment planning approach for oMRgRT, which was successfully applied to a prostate cancer patient as a first in-human experience [8, 57–60]. However, this was limited to the preplanning phase, not the adaptive phase. Integrating automatic planning into online adaptive radiotherapy, as previously explored in cone beam CT-guided radiotherapy [61], will certainly reduce treatment times and increase the access to MR-linac technology by optimizing treatment times.

## AI-driven quality assurance to ensure patient safety

Artificial intelligence-based solutions have been proposed to speed up machine quality assurance (QA) [62, 63], identifying, for example on longitudinal analyses of machine data, when failure could occur and the ideal timing for equipment service or replacement. Such techniques will likely be applicable to MR-linacs as well, though the limited number of units in operation may slow down adaptation in clinical practice, as training datasets might take longer to build.

However, the specificity of the adaptive workflow makes the application of AI even more important in the context of patient-specific quality assurance (PSQA). PSQA should validate both that that which is delivered is in line with that which was expected by the treatment planning system and also catch potential delivery errors. A growing number of studies have been published in recent years that look at the prediction of both gamma pass rates and delivery errors in standard linacs using AI-based PSQA [64]. However, for MR-guided workflows, the restrictions given by the time available for the PSQA push the requirements to the extreme.

As the online adaptation takes place while the patient is on the couch, PSQA must be efficient and should not rely on measurements, regardless of the plan complexity. Independent dose calculations based on GPUs are currently used in adaptive workflows. Several studies have tried to use deep learning to predict the result of PSQA for conventional linear accelerators [65–68] based on measurements performed at one or more institutes. Deep learning networks trained on patient data could provide an advantage compared to independent dose calculations during plan adaptation, as they may predict, from the plan features, multileaf collimator (MLC) characteristics such as shape, travel leaf motion, etc., which have been shown to be predictive for delivery accuracy [64]. Knowing such quantities—and their correlation—in advance could help to decide how to adapt the plan online, taking delivery uncertainties into account as well. Therefore, AI-based techniques may be more important for (online) adaptive workflows than for standard workflows.

The main challenge of this approach is to ensure that models for predicting such features have sufficient specificity and to maintain it over time, for example with machine upgrades and/or changes in treatment protocols. As with machine QA, data sharing and availability will be crucial, especially as the dataset will be heavily biased towards negative data. The ability of direct measurements to detect all delivery errors has been questioned [69]; therefore, the datasets will likely require input from different sources, such as independent dose calculations and in vivo dosimetry measurements, where these are available. The literature has shown that in principle, it is possible to use PSQA models derived at one institution in another institution; however, models will nevertheless need to be treatment site and energy specific, as both aspects have been shown to impact predictions [64, 66].

## AI for enhancing intrafractional motion management

Integrating an onboard MRI scanner into radiotherapy offers potential advantages for managing intrafractional motion without external surrogates and ionizing radiation. Currently, all commercially available systems use 2D fluoroscopy for image-based tracking of the target during treatment. The current commercially available systems use template matching [70, 71] or deformable image registration [72] to detect target motion. Initial studies have shown that convolutional neural networks can outperform commercially implemented tracking algorithms [73].

Current oMRgRT systems are limited to 2D dynamic MRI acquisition, thus restricting detailed 3D motion trajectory information. Advances in AI strategies are addressing this by inferring 3D motion from multislice 2D images or reconstructing 3D acquisitions, paving the way for real-time volumetric motion management [74–77]. This will enable adaptive strategies that dynamically adjust the treatment beam based on real-time motion data, leading to more precise and personalized treatments. Currently there are two options: beam gating, which is commercially available, and multi leaf collimator (MLC) tracking, which is evaluated on research systems. An in silico study by Menten et al. [78] demonstrated the feasibility of MRI-guided MLC tracking for lung SBRT. The study showed that MLC tracking significantly improves the accuracy of dose delivery, thereby allowing the use of smaller treatment margins and therefore sparing of healthy surrounding tissue. However, system latency is a concern for tracking techniques. Latency times between 104 and 347 ms [79] have been reported. Therefore, motion-prediction algorithms are needed. In the past, these algorithms were mainly based on linear regression models [80]; however, recently it was shown that AI models can outperform linear regression models for motion prediction [81, 82]. Most studies have evaluated long short-term memory (LSTM) networks for predicting motion into the future. With LSTM architecture, a prediction up to 750 ms into the future is possible [83], and even complex irregular motion can be predicted [82].

## AI-derived imaging biomarkers for outcome prediction and early toxicity assessment

MR-linacs produce imaging data as a side product that could be used to better understand the patient’s disease. Recent studies have focused on developing methods for the quantitative analysis of radiological images as prognostic and predictive imaging biomarkers, which would be fundamental to improving patient outcomes.

Van Houdt et al. [33] described in their review paper the prognostic value of quantitative MRI (qMRI) for both 0.35 T and 1.5 T magnetic field strengths. Through quantitative imaging biomarkers (QIBs) for predicting clinical outcomes and early assessment of response for decision support, qMRI is feasible for various cancer types such as head, neck, brain, sarcoma, pancreatic, and rectal cancers, with imaging available at each fraction during the treatment [84–87]. However, further development of acquisition and processing methods is required to integrate qMRI into routine clinical decision-making. Lawrence et al. [88] showed that daily quantitative diffusion-weighted imaging (DWI) through changes in the low apparent diffusion coefficient (low-ADC) regions for tumor volumes on a 1.5 T MR-linac is predictive of overall survival (OS) regions and progression-free survival (PFS). These changes in low-ADC regions also showed a higher correlation with OS and PFS compared to changes in the contrast-enhancing gross tumor volume (GTV).

Since the introduction of radiomics in the field of research, it has shown potential in identifying novel prognostic imaging-based biomarkers. The radiomic features extracted from high-quality images are analyzed mostly by correlating them with clinical outcome or local tumor control. Boldrini et al. [36] evaluated delta radiomics for the GTV in patients treated with 0.35 T MRgRT as a prediction tool in locally advanced rectal cancer. They found three representative features showing a significant correlation (*p* < 0.05) with the clinical complete response. Tomaszewski et al. [89] extracted radiomic features from the GTV of patients treated with MRgRT for pancreatic adenocarcinoma. They assessed PFS by correlating it with the ratio of values between the first and last fractions. They concluded that the ratio of the “skewness” feature had a high correlation with PFS.

Artificial intelligence may support patient-specific MR-based characterization of the tumor for prediction of treatment response or assessment of early toxicity. The AI-supported identification of imaging-based biomarkers for outcome prediction could enable tailored dose prescription depending on the early treatment response (e.g., dose escalation for nonresponsive tumor parts and/or de-escalation for early responders) after validation in clinical trials. AI may therefore facilitate the long-needed further integration of tumor biology and heterogeneity into MR-guided adaptive radiotherapy [90, 91].

Technical limitations related to imaging techniques and the resolution of scanners can affect the accuracy of image biomarker measurements. Multi-institutional comparisons showed that accuracy and short-term repeatability can be achieved by both low- and high-field MR-linacs [92, 93]. However, most uncertainty investigations have been performed in phantoms and further developments therefore are necessary. The use of deep learning might offer some advantages, but evidence based on MR-linacs is still limited [94]. The challenge with radiomics is that uncertainty arising from image acquisition, reconstruction algorithms, post-processing, and calculation of the features can affect reliability. Manual segmentation of the cancer lesions is another limitation due to intra- and interobserver variability, which has been shown to affect the feature values. To address this, Breto et al. [95] developed a deep learning strategy with a convolutional neural network for automatic glioblastoma segmentation on a 0.35 T MR-linac. This approach not only streamlines the workflow for clinicians, but also paves the way for more reliable tumor delineation and feature extraction, thereby enabling more accurate treatment outcome prediction.

## Conclusion and future outlook

The integration of AI into radiotherapy has led to significant advances over the past 5 years, particularly in the automation of key processes such as OAR delineation and treatment planning. These innovations have improved consistency, accuracy, and efficiency in clinical practice, although fully automated workflows are not yet commercially available. The introduction of MR-linacs has significantly improved treatment accuracy and the ability to adapt treatment plans in real time, particularly for tumors adjacent to radiosensitive organs. Despite these advances, MRgRT currently remains labor intensive and time consuming, highlighting the need for AI and automation to streamline workflows and support rapid decision-making. The future of radiotherapy, particularly of MRgRT, will be further transformed by AI and automation. AI algorithms will play a critical role in analyzing real-time imaging data to facilitate adaptive treatment strategies, thereby increasing precision and personalization. The generation of synthetic CTs from MR images, automated contouring, and automated treatment planning solutions will reduce reliance on manual processes, thus optimizing treatment times and widening access to MR-linac technology. In addition, AI-driven quality assurance processes will be essential to ensure patient safety by predicting potential machine errors and validating treatment delivery. Advances in intrafractional motion management enabled by AI will enable more precise and adaptive treatments. In addition, the identification and integration of imaging biomarkers for outcome prediction and early toxicity assessment will enable more tailored and effective treatment strategies.

Despite promising developments, challenges such as biases in datasets, the need for data sharing between institutions, and technical limitations in imaging techniques remain. Overcoming these challenges will be critical to the successful implementation of AI-driven solutions in clinical practice. Overall, the continued integration of AI and automation into radiotherapy promises to increase treatment accuracy, efficiency, and personalization, thus ultimately improving patient outcomes.
